# Educational technologies on sexually transmitted infections for incarcerated women

**DOI:** 10.1590/1518-8345.4365.3392

**Published:** 2020-11-06

**Authors:** Isaiane da Silva Carvalho, Tatiane Gomes Guedes, Simone Maria Muniz da Silva Bezerra, Fábia Alexandra Pottes Alves, Luciana Pedrosa Leal, Francisca Márcia Pereira Linhares

**Affiliations:** 1 Universidade Federal de Pernambuco, Recife, PE, Brazil.; 2 Universidade de Pernambuco, Faculdade de Enfermagem Nossa Senhora das Graças, Recife, PE, Brazil.; 3 Universidade Federal de Pernambuco, Departamento de Enfermagem, Recife, PE, Brazil.

**Keywords:** Educational Technology, Sexually Transmitted Diseases, Sex Education, Health Education, Women, Prisons, Tecnologia Educacional, Doenças Sexualmente Transmissíveis, Educação Sexual, Educação em Saúde, Mulheres, Prisões, Tecnología Educacional, Enfermedades de Transmisión Sexual, Educación Sexual, Educación en Salud, Mujeres, Prisiones

## Abstract

**Objective:**

to analyze in the scientific literature the educational technologies on sexually transmitted infections used in health education for incarcerated women.

**Method:**

an integrative review carried out by searching for articles in the following databases: Scopus, Cumulative Index of Nursing and Allied Health, Education Resources Information Center, PsycInFO, Medical Literature Analysis and Retrieval System Online, Latin American Literature in Health Sciences, Cochrane, and the ScienceDirect electronic library. There were no language and time restrictions. A search strategy was developed in PubMed and later adapted to the other databases.

**Results:**

a total of 823 studies were initially identified and, after applying inclusion and exclusion criteria, eight articles were selected. Most of them were developed in the United States with a predominance of randomized clinical trials. The technologies identified were of the printed materials type, isolated or associated to simulators of genital organs, videos, and games.

**Conclusion:**

the technologies on sexually transmitted infections used in health education for incarcerated women may contribute to adherence to the prevention of this serious public health problem in the context of deprivation of liberty.

## Abstract (pt)

**Objetivo:**

analisar na literatura científica as tecnologias educacionais sobre infecções sexualmente transmissíveis utilizadas na educação em saúde de mulheres encarceradas.

**Método:**

revisão integrativa realizada por meio de busca de artigos nas bases de dados Scopus, *Cumulative Index of Nursing and Allied Health, Education Resources Information Center*, PsycInFO, *Medical Literature Analysis and Retrieval System Online*, Literatura Latino-Americana em Ciências da Saúde, Cochrane e na biblioteca eletrônica *Science Direct*. Não houve restrição de idioma e tempo. Foi desenvolvida uma estratégia de busca no PubMed e, posteriormente, adaptada para as demais bases.

**Resultados:**

foram identificados inicialmente 823 estudos e após aplicação de critérios de inclusão e exclusão, selecionaram-se oito artigos. A maioria foi desenvolvida nos Estados Unidos, com predomínio de ensaios clínicos randomizados. As tecnologias identificadas foram do tipo materiais impressos, isolados ou associados a simuladores dos órgãos genitais, vídeos e jogos.

**Conclusão:**

as tecnologias sobre infecções sexualmente transmissíveis, utilizadas na educação em saúde de mulheres encarceradas, podem contribuir na adesão à prevenção desse grave problema de saúde pública no contexto da privação de liberdade.

## Abstract (es)

**Objetivo:**

analizar en la literatura científica las tecnologías educativas sobre infecciones de transmisión sexual utilizadas en la educación sanitaria para mujeres encarceladas.

**Método:**

revisión integradora realizada por medio de la búsqueda de artículos en las bases de datos Scopus, *Cumulative Index of Nursing and Allied Health, Education Resources Information Center*, PsycInFO, *Medical Literature Analysis and Retrieval System Online*, Literatura Latinoamericana en Ciencias de la Salud, Cochrane y en la biblioteca electrónica *Science Direct*. No hubo restricciones de idioma y/o fecha de publicación. Se desarrolló una estrategia de búsqueda en PubMed y, posteriormente, se adaptó a las otras bases de datos.

**Resultados:**

en un principio, se identificaron 823 estudios y después de aplicar los criterios de inclusión y exclusión, se seleccionaron ocho artículos. La mayoría se desarrollaron en los Estados Unidos, con predominio de ensayos clínicos aleatorios. Entre las tecnologías identificadas, se encuentran materiales impresos, utilizados de forma aislada o asociados con simuladores de órganos genitales, videos y juegos.

**Conclusión:**

las tecnologías sobre infecciones de transmisión sexual, utilizadas en la educación sanitaria para mujeres encarceladas, pueden contribuir en la adhesión a la prevención de este grave problema de salud pública en el contexto de la privación de libertad.

## Introduction

Sexually Transmitted Infections (STIs) are associated with more than 30 microorganisms. Of the eight most incident STIs, four are curable, namely: syphilis, gonorrhea, chlamydia, and trichomoniasis. However, hepatitis B, herpes, human immunodeficiency virus (HIV), and human papilloma virus (HPV) remain incurable despite the existence of treatment^(^ 1 ^)^.

Worldwide, more than 1 million curable STIs occur every day. In this sense, the prevention and control of these infections happen as an excellent strategy in the field of public health. In 2016, the World Health Organization launched a global initiative to reduce STIs (2016-2021). Among its principles are universal health coverage, use of evidence-based interventions, promotion of human rights with gender equality and equity in health, and empowerment of the most affected by STIs^(^ 2 ^)^.

Incarcerated women are in this group, having the STI problem enhanced during their deprivation of liberty^(^ 3 ^)^. These women are more likely to be infected with an STI when compared to the general population^(^ 4 ^-^ 6 ^)^. In addition, incarcerated individuals have a history of risky behavior in prison, such as sharing needles and unprotected sex, which favor the occurrence of this type of infection^(^ 7 ^-^ 8 ^)^. Among the STIs most associated with this population are HIV, syphilis, genital herpes, viral hepatitis, gonorrhea, chlamydia, and HPV^(^ 9 ^-^ 15 ^)^.

The Bangkok rules, an international document on the treatment of incarcerated women, state that they should receive education and information on how to prevent STIs^(^ 16 ^)^. Sometimes coming from less favored segments of society, these women have little knowledge about STI prevention^(^ 17 ^-^ 18 ^)^. This raises the need to develop health education actions with a focus on minimizing the number of cases.

Educational technologies come as a health education strategy to be considered in the teaching-learning process. These tools can, for example, stimulate lifestyle changes in the individual field, contribute to the control of risk factors considered modifiable, and favor adherence to treatments^(^ 19 ^)^.

The acquisition of new knowledge may not guarantee changes in behavior; however, in many situations, lack of knowledge can lead to inappropriate self-care behaviors. Thus, when shared among people in a concrete way, the information based on solid evidence may be able to produce changes in lifestyle regarding self-care practices in the prevention of STIs^(^ 20 ^)^.

It is highlighted that, until now, no review studies on the use of educational technologies on STIs for incarcerated women have been identified in the national and international literature. Thus, this research can contribute to fill this gap. Likewise, its results can subsidize the practice of health professionals by allowing decision-making based on scientific evidence and, at the same time, by promoting critical reflections related to the use of technologies on STIs in the perspective of health education in this population. This study aimed to analyze in the scientific literature the educational technologies on STIs used in health education for incarcerated women.

## Method

An integrative review developed based on the following stages: formulation of the problem; literature search; data evaluation; data analysis; and presentation of the results^(^ 21 ^)^. As a way of assisting data collection, we proceeded with the elaboration of a search protocol, which contained the following information: theme; objective; guiding question; search strategies (database, descriptors, and intersections); inclusion and exclusion criteria; and data collection procedure.

The research question was elaborated based on the PICo strategy: (P) - Population (incarcerated women); (I) - Interest (educational technology on STIs); (Co) - Context (health education)^(^ 22 ^)^. Thus, the following question was obtained: What are the evidences available in the literature related to educational technologies on STIs used in health education for incarcerated women?

The selected databases were Scopus, Cumulative Index of Nursing and Allied Health (CINAHL), Education Resources Information Center (ERIC), PsycInFO; MEDLINE (via PubMed), Latin American Literature in Health Sciences (*Literatura Latino-Americana em Ciências da Saúde*, LILACS), and Cochrane, in addition to the ScienceDirect electronic library. The selection of articles took place in January 2020. For that, MeSH controlled descriptors and their synonyms were used. An asterisk was added to the descriptors to retrieve studies that presented words stemming from the same radical. The descriptors were combined using the Boolean operators “AND” and “OR”. Articles indexed with MeSH terms were selected, as well as their presence or their synonyms in the title/abstract. Initially, the search strategy was developed in PubMed and was later adapted for the other databases and electronic library (Figure 1).

**Figure 1:** PICo strategy and descriptors used. Recife, PE, Brazil, 2020

PICo strategy*		
P (Population)	**Incarcerated women**	List of descriptors
		**Women** [MH] Woman [TIAB] Girl* [TIAB] **Female** [MH] Female* [TIAB] **Prisons** [MH] Prison* [TIAB] Incarceration [TIAB]
I (Interest)	**Educational technology on STIs^†^**	**Educational Technology** [MH] Educational Technolog* [TIAB] Instructional Technolog* [TIAB] **Sexually Transmitted Diseases** [MH] Sexually Transmitted Diseases [TIAB] STIs [TIAB] Venereal Diseas* [TIAB] **Sexually Transmitted Infections** [MH] Sexually Transmitted Infection* [TIAB] STDs [TIAB]
Co (Context)	**Health education**	**Health Education** [MH] Health Education [TIAB] **Health Promotion** [MH] Health Promotion [TIAB] Community Health Education [TIAB] **Sex Education** [MH] Sex Education [TIAB]
**Search strategy in PubMed**		
1. Women[MeSH Terms] OR Woman[Title/Abstract] OR Girl*[Title/Abstract] OR Female[MeSH Terms] OR Female*[Title/Abstract] AND Prisons[MeSH Terms] OR Prison*[Title/Abstract] OR Incarceration[Title/Abstract] 2. Educational Technology[MeSH Terms] OR Educational Technolog*[Title/Abstract] OR Instructional Technolog*[Title/Abstract] AND Sexually Transmitted Diseases[MeSH Terms] OR Sexually Transmitted Diseases[Title/Abstract] OR STIs[Title/Abstract] OR Venereal Diseas*[Title/Abstract] OR Sexually Transmitted Infections[MeSH Terms] OR Sexually Transmitted Infection*[Title/Abstract] OR STDs[Title/Abstract] 3. Health Education[MeSH Terms] OR Health Education[Title/Abstract] OR Health Promotion[MeSH Terms] OR Health Promotion[Title/Abstract] OR Community Health Education[Title/Abstract] OR Sex Education[MeSH Terms] OR Sex Education[Title/Abstract] 4. #1 AND #2 AND #3		

* PICo = Population, interest, and context;

† STIs = Sexually transmitted infections

The adopted inclusion criteria were the following: original articles that addressed the use of educational technology for incarcerated women, published until 2019 in any language and available electronically in full. Articles that did not answer the research question were excluded.

The databases were accessed through the journal portal of the Coordination for the Improvement of Higher Level Personnel, via institutional remote access. This procedure was adopted to expand the search for articles in their entirety. For data extraction, a script was prepared in Excel containing the following information: author, title, year of publication, country, language, journal, objective, study design, number of participants, type of technology, type of STI, outcome, and level of evidence.

For the classification of the level of evidence, the following division was adopted: 1A - Systematic review of randomized controlled clinical trials; 1B - Randomized controlled clinical trial with a narrow confidence interval; 1 - Therapeutic results of the “all or nothing” type; 2A - Systematic review of cohort studies; 2B - Cohort study (including randomized clinical trial of lesser quality); 2C - Observation of therapeutic results and ecological study; 3A - Systematic review of case-control studies; 3B - Case-control study; 4 - Case report (including lower quality cohort or case-control); and 5 - Expert opinion^(^ 23 ^)^.

The articles were exported to the *EndNote* online program to remove duplicate studies. Then, the titles and abstracts of the articles were examined in *EndNote* itself and those that met the selection criteria were considered for the next phase. These were organized according to the selection database/library, in an Excel spreadsheet. Subsequently, the article was read in full. The selection was carried out independently by two researchers, and the differences were solved by consensus.

Data analysis was performed in a descriptive manner with the results presented in a summary table and discussed based on the available literature on the subject. As the study did not involve research with human beings, there was no need for submission to the Research Ethics Committee. However, it is highlighted that the authors’ original ideas were maintained when proceeding with the synthesis of the results. The Preferred Reporting Items for Systematic Reviews and Meta-Analyses (PRISMA)^(^ 24 ^)^ recommendations were adopted for drafting the manuscript.

## Results

**Figure 2:**
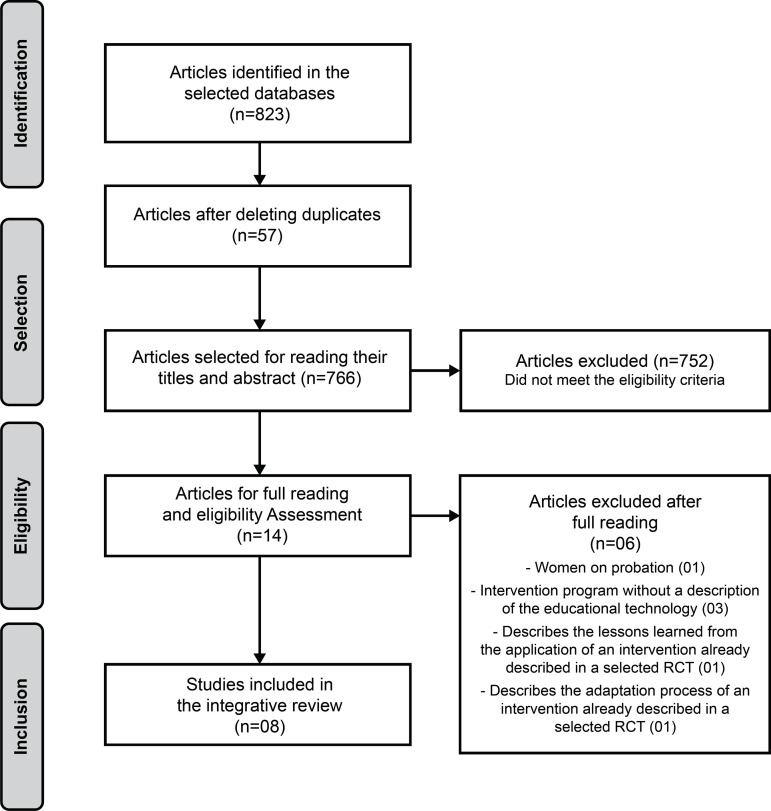
Flow of article selection process Recife, PE, Brazil, 2020

Source: Adapted from the Preferred Reporting Items for Systematic Reviews and Meta-Analyses **(PRISMA)**^(^24^)^ flow diagram

A total of 823 articles were identified and eight were selected after applying the inclusion and exclusion criteria, as shown in Figure 2.

Of the eight studies that were included in the integrative review, five were identified in MEDLINE^(^ 25 ^-^ 29 ^)^, one in Cochrane^(^ 30 ^)^, one in ScienceDirect^(^ 31 ^)^, and one in CINAHL^(^ 32 ^)^. Most of the studies were developed in the United States (7)^(^ 25 ^-^ 30 ^,^ 32 ^)^ and all of them were written in English. Only one article was prepared in Brazil^(^ 31 ^)^. Publications between 1997 and 2018 were identified, with a predominance of 2015, which presented four publications^(^ 26 ^,^ 30 ^-^ 32 ^)^. Each article was published in a different journal. Only two articles were published in journals linked to the areas of behavioral and social sciences^(^ 31 ^)^ and interpersonal violence^(^ 32 ^)^. The others were associated with the health area. As for the type of study, five were randomized clinical trials^(^ 25 ^-^ 26 ^,^ 30 ^-^ 32 ^)^ and three were descriptive studies^(^ 26 ^,^ 31 ^-^ 32 ^)^.

The educational technologies used on STIs for incarcerated women were printed materials with two interventions with this type of material associated with genital organ simulators^(^ 31 ^-^ 32 ^)^, followed by video^(^ 28 ^-^ 30 ^)^ and games^(^ 27 ^)^. It should be noted that the educational technologies described were not used in isolation, but as part of an educational intervention that involved, for example, group^(^ 26 ^,^ 29 ^,^ 31 ^)^ sessions or combinations of group and individual sessions^(^ 25 ^,^ 32 ^)^. Regarding the types of STIs, HIV was the object of two technologies^(^ 26 ^,^ 28 ^)^ and one study addressed HIV and the Hepatitis C virus^(^ 25 ^)^. The other studies addressed STIs/HIV^(^ 27 ^,^ 29 ^-^ 32 ^)^. As for the level of evidence, one randomized clinical trial was classified as 1B^(^ 28 ^)^, and four as 2B^(^ 25 ^,^ 27 ^,^ 29 ^-^ 30 ^)^. The other studies were classified as having a level of evidence of 4^(^ 26 ^,^ 31 ^-^ 32 ^)^. A chart was created to summarize the main characteristics of the studies (Figure 3).

**Figure 3:** Presentation of the articles included in the review. Recife, PE, Brazil, 2020

Authors, year of publication and country	Objective	Type of study and No. of participants	Educational technology	Evidence level
Staton, et al., 2018^(25)^ United States	To evaluate a standard educational intervention and an improved individualized intervention (standard educational intervention + motivational interview) to reduce the risk of HIV* in women who use drugs in rural prisons.	Randomized clinical trial n=400	Printed material (cards)	2B
Fogel, et al., 2015^(30)^ United States	To test the effectiveness of an adapted, evidence-based behavioral intervention for the prevention of HIV*/STDs^†^ among incarcerated women.	Randomized clinical trial n=521	Video	2B
Guedes, et al., 2015^(31)^ Brazil	To carry out educational actions focused on preventing the transmission of sexual diseases/human immunodeficiency virus, safe sexual practices, family planning, violence, and prevention of uterine and breast cancer.	Descriptive study n= not informed	Printed material (photos and pictures) + simulators of genital organs	4
Gupta, et al., 2015^(26)^ United States	To describe the program, the baseline, and the characteristics of the participating women; and to present the results of the evaluation of the program called *Tudo sobre profilaxia pós-exposição não ocupacional* (Everything about non-occupational post-exposure prophylaxis).	Descriptive study n=114	Printed material (folder)	4
Johnson, et al., 2015^(32)^ United States	To report the justification for the intervention, feasibility, acceptability, and pre-post results in a small initial feasibility study conducted with 14 women who received the intervention before release from prison, with follow-up assessments at 2, 5, and 8 months after release.	Descriptive study n=14	Printed material (safe sex booklet) + simulators of genital organs	4
DiClemente, et al., 2014^(27)^ United States	To assess the effectiveness of an intervention to reduce STD^†^ incidents, improve HIV* preventive behaviors, and improve the psychosocial outcomes.	Randomized clinical trial n=188	Interactive computer games	2B
Knudsen, et al., 2014^(28)^ United States	To evaluate the *Reducing Risky Relationships* intervention for HIV * in a randomized multi-site clinical trial.	Randomized clinical trial n=444	Video	1B
Lawrence, et al., 1997^(29)^ United States	To compare an intervention based on the social cognitive theory with a comparison condition based on the gender theory and the power in reducing the risk of HIV* in incarcerated women.	Randomized clinical trial n=90	Video	2B

* HIV = Human immunodeficiency virus;

† STDs = Sexually transmitted diseases

## Discussion

The technologies identified in this review used in health education about STIs for incarcerated women were printed materials, used alone or associated with simulators of genital organs, videos, and games. The absence of other types of technologies, especially the ones that use the Internet, can be a consequence of the difficulties of their insertion in the context of incarceration due to institutional security issues. This condition represents a challenge for the researchers in the area and, at the same time, encourages the development of alternative strategies to the limitations imposed by the deprivation of liberty scenario.

Regarding the period in which the studies were published, there is little expression of the use of this type of educational resource in the 20^th^century^(^ 29 ^)^. On the other hand, since 2014 there has been a significant increase in the description in scientific studies of the use of technologies in the health education process related to the STIs for women deprived of their liberty^(^ 25 ^-^ 28 ^,^ 30 ^-^ 32 ^)^. This may reflect the growing number of studies aimed at building technologies developed to improve the health care and education process. In this sense, in view of the magnitude of the STI problem in the prison context, it is expected that in the coming years new technologies can be produced to assist the activities developed by the professionals in these areas and their effect assessed through robust studies.

In addition, the period of conducting new studies corresponds to the period of increase in the number of women incarcerated in the world. There has been a growth of more than 50% of women deprived of their liberty worldwide since 2000, with values that exceed 700,000^(^ 33 ^)^. Added to this are the changes produced in the field of health education and teaching with the insertion of new technologies.

However, the use of digital technologies or equipment does not correspond to an innovation in teaching from a methodological point of view. When applied in the field of education, these resources can be an aid tool in the teaching and learning process. It is necessary that the purposes for their use are clear. In addition, the use of educational technologies requires adaptations for those involved in this process, including the environment^(^ 34 ^)^.

The fact that the majority of the studies have been carried out in the United States shows the importance given by the scientific community to the problem of STIs for incarcerated American women. At the same time, it reflects the extent of the problem, since the United States is the country with the largest population of women deprived of their liberty in the world, with more than 200,000^(^ 33 ^)^.

The health demands of this population are complex, which is enhanced by the low visibility in the USA public policies. As a consequence, fewer resources are allocated when compared to the male prison population, which results in the inability of the prison institutions to respond to the women’s health needs^(^ 35 ^)^. This reality is aggravated when considering the panorama of underdeveloped countries that face numerous problems in sensitive social sectors such as health, education, and security, and which have to deal with a limited amount of financial resources.

Regarding the language, it is understandable that, as English is the main language used by the scientific community to disseminate research studies^(^ 36 ^)^, it has had an absolute presence. STIs are considered a serious public health problem^(^ 1 ^)^ and this explains the fact that the studies have been published especially in health journals. However, this is a problem that, when associated with incarceration, can be dealt in a cross-sectional manner, which justifies the presence of articles published in other areas of knowledge^(^ 31 ^-^ 32 ^)^.

The research studies of the randomized clinical trial type were the main study designs adopted by the authors and showed that the interventions performed were tested following a method capable of producing robust evidence. The results from studies of this nature represent one of the best evidence available in the scientific community, given the rigor with which they are conducted^(^ 37 ^)^. This reflects the high level of evidence obtained by most of the studies, although among the clinical trials, only 1 has obtained level 1B. This occurred due to the follow-up losses greater than 20% present in the other trials, which makes them be classified as 2B^(^ 23 ^)^.

The interventions in which the technologies described above were used varied as to the manner of employment, making evident a broader process that involved group and individual sessions^(^ 25 ^-^ 26 ^,^ 29 ^,^ 31 ^-^ 32 ^)^ and in, some cases, even follow-up after freedom was granted^(^ 27 ^-^ 28 ^,^ 30 ^)^. This reinforces the concept that technology is another tool to assist the professionals in the health education process and that the results obtained cannot be attributed exclusively to their employment, but to the intervention as a whole.

Regarding the STIs, there was a strong presence of HIV/AIDS. The prevalence of HIV in prisons is higher than in the community^(^ 38 ^)^. In this sense, fighting HIV infection in prison settings represents an enormous challenge for both the health and the judiciary system. In addition, it is necessary to consider the repercussions that HIV infection can cause in both the individual and collective fields. Likewise, the costs associated with prevention are notably lower than those spent on treatments^(^ 39 ^)^.

The printed material was frequently used as educational technology in the studies analyzed, sometimes associated with other technologies or strategies, such as simulators of genital organs^(^ 31 ^-^ 32 ^)^. It is known that the prison setting has limitations regarding the entry of technological resources, even if educational. Digital technologies or those that need access to the Internet are hardly used in this setting. This is related to the choice of printed materials, such as folders, pictures, photos, and booklets^(^ 31 ^-^ 32 ^)^.

Other resources such as videos and games were also used^(^ 27 ^-^ 30 ^)^. These technologies can be built and used to boost the teaching and learning process without the need for Internet access, which favors their use in prison settings.

Videos are educational technologies that can be used in health education, either in isolation or in association with other technologies^(^ 40 ^)^. In the context of the STIs, brief interventions based on videos can be considered economical tools in the prevention of new cases, especially in places where the time of the health professionals is limited or where prevention programs with a longer duration are not available^(^ 41 ^)^.

As for the use of games, these allow the student to memorize information, stimulate learning and greater involvement, conditions that favor its use at different moments in the teaching-learning process^(^ 42 ^)^. The game can be used as a prevention and continuous care strategy for specific diseases, such as HIV. Thus, by means of an interactive and dynamic approach, important behavioral changes related to health can be achieved^(^ 43 ^)^.

Regardless of the technology to be used, it is important to consider active methods in the health education process and to understand that their use is more than the simple insertion of a technological resource^(^ 44 ^)^. A systematic review with meta-analysis showed that active learning methods promoted better performance of underrepresented students (ethnic, racial, and low-income minorities) in science, technology, engineering, and mathematics courses when compared to traditional classes^(^ 45 ^)^.

Finally, most of the studies have highlighted the importance of these interventions as a viable strategy for reducing risky behaviors and for the consequent STI prevention in incarcerated women. Such results can provide subsidies for nurses and other health professionals with regard to the development of new technologies and educational programs associated with STIs in the female prison context.

As a limitation, there is the fact that most of the studies correspond to the North American reality, which presents particularities regarding the profile of the population of incarcerated women, the health system, and justice.

## Conclusion

This review concluded that the educational technologies on sexually transmitted infections most used for incarcerated women were printed materials, either isolated or associated with simulators of genital organs, videos, and games. These technologies were described with a predominance of the approach on HIV/AIDS, mainly in studies such as randomized clinical trials, which confirms the importance of this problem among the infections that affect incarcerated women. Thus, the use of these technologies from the perspective of health education may contribute to the prevention of this serious public health problem in the context of deprivation of liberty.

In terms of knowledge gaps, it was observed that some studies did not describe the validation process of the educational technologies or programs used. In addition, the lack of studies in other parts of the world compromises the generalization of the results and, when present, the short follow-up time makes it difficult to measure the impact of the intervention in the long term.

New studies need to be developed to assess the effect of these technologies, especially in other countries, such as Brazil. Likewise, other technologies on STIs prevalent in this population need to be produced, taking into account a solid validation process and the particularities present in the prison system.
